# Serologic host response to *Helicobacter pylori* and *Campylobacter jejuni* in socially housed Rhesus macaques (*Macaca mulatta*)

**DOI:** 10.1186/1757-4749-4-9

**Published:** 2012-08-24

**Authors:** Sabine Kienesberger, Guillermo I Perez-Perez, Juan L Rivera-Correa, Rafael Tosado-Acevedo, Huilin Li, Andre Dubois, Janis A Gonzalez-Martinez, Maria Gloria Dominguez-Bello, Martin J Blaser

**Affiliations:** 1 Department of Medicine, NYU Langone Medical Center, New York, NY, USA; 2 Department of Microbiology, NYU Langone Medical Center, New York, NY, USA; 3 VA Medical Center, New York, NY, USA; 4 Department of Population Health, NYUSOM, New York, NY, USA; 5 Department of Biology, University of Puerto Rico, San Juan, PR, USA; 6 Interamerican University of Puerto Rico, Metropolitan Campus, San Juan, PR, USA; 7 Digestive Diseases Division, Department of Medicine, Uniformed Services of the Health Sciences, Bethesda, MD, USA; 8 Caribbean Primate Research Center, University of Puerto Rico-Medical Sciences Campus, San Juan, PR, USA

**Keywords:** *Helicobacter pylori*, *Campylobacter jejuni*, Rhesus macaques, Antibodies, Sero-prevalence, CagA

## Abstract

**Background:**

*Helicobacter pylori* are successful colonizers of the human gastric mucosa. Colonization increases the risk of peptic ulcer disease and adenocarcinoma. However, potential benefits of *H. pylori* colonization include protection against early-onset asthma and against gastrointestinal infections. *Campylobacter jejuni* are a leading cause of bacterial diarrhea and complications include Guillain-Barré syndrome. Here, we describe the development of reliable serological assays to detect antibodies against those two bacteria in Rhesus macaques and investigated their distribution within a social group of monkeys.

**Methods:**

Two cohorts of monkeys were analyzed. The first cohort consisted of 30 monkeys and was used to establish an enzyme-linked immunosorbent assay (ELISA) for *H. pylori* antibodies detection. To evaluate colonization of those macaques, stomach biopsies were collected and analyzed for the presence of *H. pylori* by histology and culture. *C. jejuni* ELISAs were established using human serum with known *C. jejuni* antibody status. Next, plasma samples of the 89 macaques (Cohort 2) were assayed for antibodies and then statistically analyzed.

**Results:**

An *H. pylori* IgG ELISA, which was 100% specific and 93% sensitive, was established. In contrast, the IgA ELISA was only 82% specific and 61% sensitive. The CagA IgG assay was 100% sensitive and 61% of the macaques were positive. In cohort 2, 62% macaques were *H. pylori* sero-positive and 52% were CagA positive. The prevalence of *H. pylori* IgG and CagA IgG increased with monkey age as described for humans. Of the 89 macaques 52% showed IgG against *C. jejuni* but in contrast to *H. pylori,* the sero-prevalence was not associated with increasing age. However, there was a drop in the IgG (but not in IgA) mean values between infant and juvenile macaques, similar to trends described in humans.

**Conclusions:**

Rhesus macaques have widespread exposure to *H. pylori* and *C. jejuni,* reflecting their social conditions and implying that Rhesus macaques might provide a model to study effects of these two important human mucosal bacteria on a population.

## Background

*Helicobacter pylori* are Gram-negative bacteria that colonize the gastric mucosa of humans across the world. However, *H. pylori* is disappearing from populations in developed countries [1,2]. In developing countries, up to 90% of the adult population carries the organism [3,4]. *H. pylori* is acquired early in life [5,6] and generally persists unless hosts are treated with antibiotics [1]. Gastric *H. pylori* colonization increases risk of peptic ulcer disease as well as adenocarcinoma of the distal stomach [7]. In addition to negative effects late in life, there is now evidence that *H. pylori* may protect against early-onset asthma [8-10] and gastrointestinal infections [11-13], thus providing benefits early in life. Because Rhesus macaques usually are persistently colonized with *H. pylori* and develop chronic gastritis [13,14], they represent a model to study host interactions.

*Campylobacter jejuni* are Gram-negative bacteria that are among the leading causes of acute gastroenteritis worldwide [15]. Sequelae of *C. jejuni* infections may include the Guillain–Barré syndrome and reactive arthritis [16-18]. *C. jejuni* infections are known to be highly prevalent within monkey colonies, especially when the animals are living under non-natural conditions [19]. Despite recent advances [20-24], the colonization dynamics of *H. pylori* and *C. jejuni* in macaques in relation to infection in humans have been little examined. The purpose of this study was to (I) establish reliable serological assays to detect monkey antibodies to *H. pylori-* and *C. jejuni*-specific antigens and (II) to investigate the sero-prevalence of *H. pylori* and *C. jejuni* in a social-group of Rhesus macaques. We hypothesized that a group of monkeys with constant contact with each other would be manifested by a high prevalence of responses to these enteric organisms, similar to those shown by humans before the introduction of antibiotics and better hygienic standards. Such assays could provide models to study *H. pylori* spread, eradication, and putative positive and negative effects in individuals and in populations.

## Results

### Verification of ELISA for determination of *H. pylori* sero-status using Rhesus macaque Cohort 1

Cohort 1 was used to establish reliable cut-off values for *H. pylori* sero-positivity to analyze Cohort 2. According to endoscopy performed on the 30 animals in Cohort 1, 13 macaques were negative for *H. pylori* and 17 were positive. Specific plasma IgG to *H. pylori* was substantially higher in animals who had been shown by endoscopy to be colonized compared to negative macaques (Table 1). Having a positive IgG antibody (ODR >0.340) determination was 100% sensitive but it was only 70% specific for colonization when endoscopy was used as the gold standard (Table 2). Because of presumed falsely negative endoscopic results observed in the initial samples, we defined *H. pylori*-positivity by either a positive endoscopy or IgG ODR >0.340 on the initial plasma. When we tested these combined criteria for 40 follow-up plasma obtained from the same animals, we found that this combination was highly accurate (93% sensitive, 100% specific). Four macaques were *H. pylori* biopsy-negative on the initial examination but had high CagA, IgG, and IgA values as well as high gastric inflammation scores (Table 1). The CagA assay was highly specific (100%) and 61% of the *H. pylori*-positive macaques were CagA-positive, which is similar to the prevalence in humans [25-27]. In contrast, the IgA ELISA was only 82% specific and 61% sensitive (Table 2). In total, we conclude that determination of the *H. pylori* IgG status is highly accurate in Rhesus macaques, reflecting the actual *H. pylori* colonization status. As such, we could use it to assess *H. pylori* status in monkeys without endoscopy.

**Table 1:** ***H. pylori *antibody responses and inflammation scores for the 30 Rhesus macaques of Cohort 1**

**Evidence for**		**Mean ± SD**^***a***^				
***H. pylori* presence**		***H. pylori***			**CagA**		**Inflammation score**
**Endoscopy**	**IgG serology**	**n**	**IgG**	**IgA**	**IgG**		
-	-	9	0.13 ± 0.07	0.20 ± 0.12	0.08 ± 0.06	1.25 ± 1.39
-	+	4	0.75 ± 0.27	0.66 ± 0.45	0.41 ± 0.24	2.25 ± 0.96
+	+	17	1.10 ± 0.38	0.94 ± 0.53	0.71 ± 0.50	4.56 ± 1.83

^*a*^ Mean ODR-values were significantly (p < 0.001) different between the known negative (n = 9) and the known positive (n = 17) monkeys for each antibody response and for inflammation score. Mean values were significantly different between the negative monkeys and the monkeys that were endoscopy negative but IgG positive (n = 4) for CagA antibody response (p < 0.02) but not for inflammation score.

**Table 2:** **Serological responses to *H. pylori *antigens in initial and follow-up sera from Cohort 1 Rhesus macaques in relation to initial *H. pylori *status**

			**% positive**		
			***H. pylori***		**CagA**
**Timing**	**Status**	**Number of specimens**	**IgG**	**IgA**^***c***^	**IgG**^***d***^
Initial	+^*a*^	17	100	88	82
	-	13	30	23	23
Follow-up	+^*b*^	28	93	61	61
	-	12	0	18	0

^*a*^ Positive (colonized) defined as detection of *H. pylori* from culture or histological examination of gastric antral biopsy.

^*b*^ Positive (colonized) defined as detection of *H. pylori* from culture or histological examination of gastric antral biopsy, or presence of IgG ODR (>0.34) in the initial specimen. A total of 40 follow-up serum specimens were available for examination.

^*c*^ Positive was defined as ODR >0.4; based on Mean + 2 intervals of SD of values from reference group of uninfected monkeys.

^*d*^ Positive was defined as ODR >0.2; based on Mean + 2 intervals of SD of values from reference group of uninfected monkeys.

### Cohort 2: Sero-prevalence of *H. pylori* is higher in older Rhesus macaques

**Figure 1:**
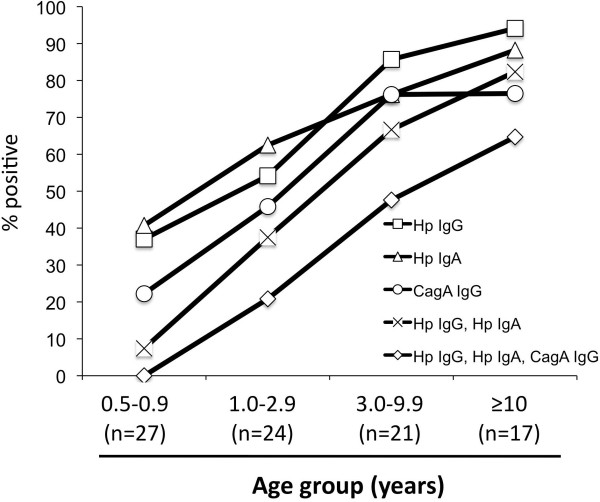
***H. pylori *IgG, IgA, and CagA status of 89 healthy Rhesus macaques by age.** Rates of positivity for *H. pylori *colonization and CagA-status, according to five different serological classifications defining positivity.

First, we examined the ODR-values obtained for the Cohort 2 monkeys. Since the cut-offs obtained from the Cohort 1 macaques and from Cohort 2 were very similar (Table 3), we considered them to be reliable. We then examined the *H. pylori* IgG status in 89 macaques in Cohort 2, using the determined cut-off. Summaries of the data are shown in Table 4 and Figure 1. A total of 58 (62%) of the 89 Rhesus macaques were *H. pylori* sero-positive and the sero-prevalence of *H. pylori* increased with age (Cochran-Armitage Trend Test, p < 0.0001).

**Table 3:** **Summary of threshold cut-off values in groups of Rhesus macaques of unknown *H. pylori *status**

	***H. pylori***		**CagA**
**Antibody**	**IgG**	**IgA**	**IgG**
Cut-off from Cohort 1	0.340	0.440	0.200
Negative (0.000-0.299)^*b*^	0.344	0.363	0.198
Positive (0.400-1.000)^*b*^	0.297	0.362	0.299
**Mean Value**^***a***^	**0.327 (0.340)**	**0.388 (0.400)**	**0.232 (0.200)**
Standard Deviation	0.024	0.057	0.050

^***a***^ Values in ( ) describe the actual ODR-cut-off values used to analyze Cohort 2.

^*b*^ As calculated, for Cohort 2.

**Table 4:** ***H. pylori *positivity of 89 Rhesus macaques by age**^***a***^

**Group 1 – Infants (0.5-0.9 years) (n = 27)**						
	IgG + ^*b*^	CagA+	IgG+/CagA+	IgG+/cagA-	IgG-/CagA+	IgG-^*c*^
Total #	10	6	2	5	4	17
**%**	**37**	**22**	**7**	**16**	**15**	**63**
**Group 2 – Juvenile (1.0-2.9 years) (n = 24)**						
	IgG + ^*b*^	CagA+	IgG+/CagA+	IgG+/cagA-	IgG-/CagA+	IgG-^*c*^
Total #	13	11	8	2	3	11
**%**	**54**	**46**	**33**	**8**	**13**	**46**
**Group 3 – Young adult (3.0-9.9 years) (n = 21)**						
	IgG + ^*b*^	CagA+	IgG+/CagA+	IgG+/cagA-	IgG-/CagA+	IgG-^*c*^
Total #	18	16	13	2	3	3
**%**	**86**	**76**	**62**	**10**	**14**	**14**
**Group 4 – Adult (≥10 years) (n = 17)**						
	IgG + ^*b*^	CagA+	IgG+/CagA+	IgG+/cagA-	IgG-/CagA+	IgG-^*c*^
Total #	16	13	13	3	0	1
**%**	**94**	**76**	**76**	**18**	**0**	**6**
**Total (n = 89)**						
	IgG + ^*b*^	CagA+	IgG+/CagA+	IgG+/cagA-	IgG-/CagA+	IgG-
Total #	57	46	36	12	10	32
**%**	**64**	**52**	**40**	**13**	**11**	**36**

^*a*^ From the total of 94 Rhesus macaques studied, 5 that were <0.5 years of age were excluded.

^*b*^ positive in either the *H. pylori* or CagA IgG assay.

^*c*^ negative in both the *H. pylori* and CagA IgG assays.

### *H. pylori* IgA status correlates with the IgG status only in older Rhesus macaques

The serum of the 89 animals was also tested for the presence of serum IgA antibodies to *H. pylori* antigens using the IgG-determined status as a standard. An IgA cut-off at 0.39 was considered predictive to determine the *H*. *pylori* status since it yielded the same percent (64%) of positivity. Of the 57 macaques determined to be IgA-positive (Figure 1), in the youngest group, only 2 (7%) monkeys were both IgA and IgG positive, but 9 (38%) group 2 monkeys, 14 (67%) group 3 monkeys and 14 (82%) monkeys of the oldest group showed double-positive status (Figure 1). Thus, IgG antibodies become more prevalent with age in the *H. pylori*-positive monkeys.

### CagA sero-prevalence correlates with *H. pylori* IgG status

When the Rhesus macaques of Cohort 2 were analyzed with the CagA cut-off established at 0.200, 52% of the macaques were CagA+, similar to the 61% of CagA + monkeys of Cohort 1. As with the *H. pylori* IgG, the prevalence of CagA antibodies increased with the age of the monkeys (Table 4 and Figure 1) (Cochran-Armitage Trend Test, p < 0.0001). With the exception of the youngest group of Rhesus macaques, the *H. pylori* status closely correlated with the CagA status. Next, we compared the percent of macaques positive for both *H. pylori* and CagA with those that only were positive for *H. pylori* (Table 4). The ratio of double-positive monkeys increased with age (Figure 1). In total, 36 (40%) of the 89 animals were positive in both assays and 12 (13%) were only positive for *H. pylori*.

### *H. pylori* IgG and IgA ODR-values and CagA IgG ODR-values are higher in older Rhesus macaques

**Figure 2:**
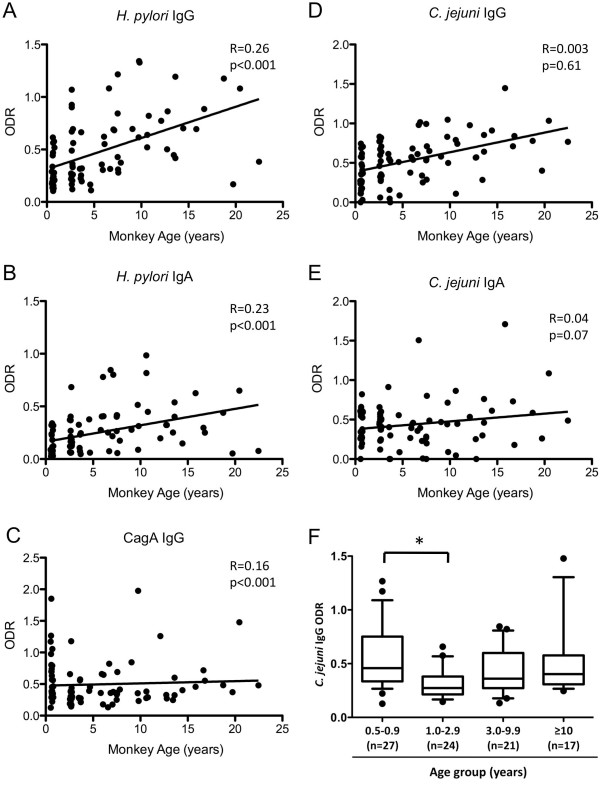
**Linear regression analysis of *H. pylori *and *C. jejuni *serum antibodies by monkey age.** ODR-values for *H. pylori *IgG and IgA and CagA IgG (Panels **A-C**). Outliers (p < 0.001) were excluded from analysis in the *H. pylori *IgG group (n = 3); R-values and p-values are provided in each panel. For *H. pylori *IgG, *H. pylori *IgA, CagA IgG, N = 89. *H. pylori* serum antibody levels were positively correlated with age (p < 0.001, in each case). *C. jejuni* IgG and IgA ODR-values in relation to monkey age (Panels **D-E**). *C. jejuni* IgA and IgG, N = 89; For *C. jejuni*, older age was not associated with higher ODR-values (p > 0.05). Analysis of *C. jejuni *IgG median ODR-values (Panel **F**). For the box-plot analyses (Median 25–75 percentiles shown, with 10–90 percentile shown as whiskers. Outliers (p < 0.001) were excluded. * p = 0.035.

If our analysis is correct and sero-prevalence is truly higher in older monkeys, ODR-values and monkey age should be correlated. Linear regression analysis to examine *H. pylori* IgG and IgA and CagA IgG values in relation to monkey age were performed (Figure 2A-C). For both *H. pylori* IgG and IgA, there were significant trends of higher values with age. The same relationship was obtained for CagA IgG. This remains true after adjusting for gender in the linear regression analysis. Moreover, *H. pylori* IgG levels in female monkeys appear to increase faster as they age compared with males values. Females also had elevated *H. pylori* IgA and CagA IgG levels compared to males throughout their lifetime (not shown). There also were significant gender differences in *H. pylori* IgG sero-prevalence between females and males (Odd ratio = 2.56, 95% CI: 1.04-6.32, p = 0.04) when the total number of monkeys was analyzed. In summary, older Rhesus macaques have higher *H. pylori* IgG and IgA and CagA IgG ODR-values, consistent with a higher prevalence of *H. pylori* and of CagA-positive strains in older monkeys.

### CagA status of IgA/IgG positive Rhesus macaques

Using a very stringent criterion in which only IgA and IgG double-positive macaques were considered as *H. pylori*-positive (Figure 1), there were 39 (44%) monkeys that were double-positive. Of these, 26 (67%) were triple-positive (*H. pylori* and CagA IgG, and *H. pylori* IgA).

### *C. jejuni* sero-prevalence is not associated with increasing Rhesus macaque age

**Figure 3:**
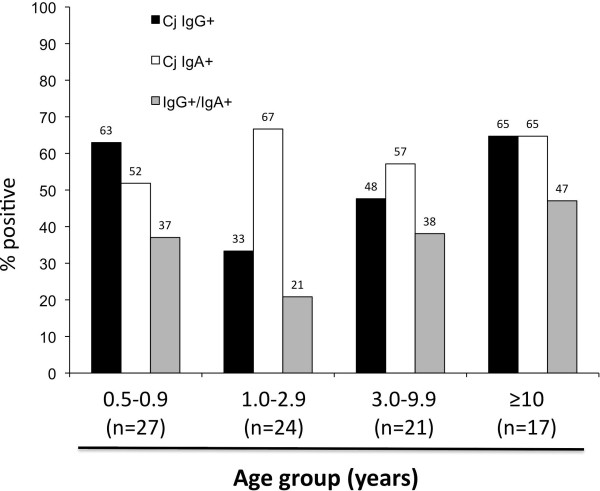
***C. jejuni *sero-status of 89 healthy Rhesus macaques by age.**

We also analyzed *C. jejuni* positivity by using a cut-off value obtained by both testing 26 human samples of known *C. jejuni* status [28], and by using statistical evaluation of Cohort 2 values. A total of 59 monkeys (52%) showed serum IgG against *C. jejuni* (Figure 3). There was no correlation between age and infection status (p = 0.23). Using stringent criteria, counting only IgA and IgG double-positive monkeys as *C. jejuni* positive (Figure 3), a total of 31 monkeys (35%) were thus positive. The IgA and IgG status were not correlated, and *C. jejuni* sero-positivity also was not age-related (Cochran-Armitage Trend Test, p = 0.79). There was no positive or negative association at any age between *H. pylori* and *C. jejuni* IgG status.

### *C. jejuni* IgG and IgA ODR-values do not increase with Rhesus macaque age

As described for *H. pylori*, using regression analysis, we determined whether IgG and IgA ODR-values change with age. In contrast to *H. pylori*, *C. jejuni* IgG or IgA values are not higher in older macaques (Figure 2D-E). When *C. jejuni* IgG mean values for each group were analyzed separately, there was a significant difference (p = 0.004) between groups 1 and 2, but there was no drop for IgA (Figure 2F). A similar trend has been described in humans [29,30]. The IgA median stayed constant for the first three groups and rose in the oldest Rhesus macaques. In contrast to *H. pylori* colonization, *C. jejuni* positivity did not substantially increase with age.

## Discussion

The original habitats of Rhesus macaques range from Central to Southeast Asia [31]. However, populations of Rhesus macaques have been living in captivity for generations, since they are the most important non-human primates used for medical and biological research [32-36]. For this study, we analyzed gastric biopsies and blood samples from the animal facility at the Uniformed Services University of the Health Sciences, to establish reliable cut-off values for detecting *H. pylori* antibodies (Cohort 1). To investigate the sero-prevalence of responses to *H. pylori* and *C. jejuni* antigens in social Rhesus macaques, we studied blood samples routinely taken from a population of animals living together in one outdoor cage in the Caribbean Primate Research Center since 1984 (Cohort 2). We sought to determine how prevalent *H. pylori* and *C. jejuni*-specific antibodies are in a group of Rhesus macaques to evaluate whether the colonization rates of monkeys are similar to human populations.

Although *H. pylori* and *C. jejuni* are phylogenetically close, they differ substantially in the nature of their antigens [37], even including conserved proteins like their heat-shock protein 60 [38]. Among other factors, flagellae proteins [39], adhesins and outer membrane proteins differ considerably between *C. jejuni* and *H. pylori*[40]. In this study we used water-extracted proteins as antigens. Potential cross-reactions between *H. pylori* and *Campylobacter* antigens have been addressed in previous studies [41,42] and are considered to be of minor significance [43]. Moreover, the immune response to CagA is specific for *H. pylori* and no cross-reactions have been described.

Using the biopsy and blood samples from Cohort 1, we were able to establish ELISAs that were 100% specific and 93% sensitive for *H. pylori* IgG detection, and 100% sensitive for CagA IgG. In contrast, the IgA ELISA was much less specific and sensitive and as is the case with humans [44], not sufficiently accurate for classification of *H. pylori* status in Rhesus macaques.

The determined ELISA parameters were used to evaluate Cohort 2 for *H. pylori* prevalence. In developed countries, *H. pylori* is disappearing [1,2] but in developing countries, *H. pylori* prevalence remains high [3,4]. The prevalence of *H. pylori* in monkeys clearly increases with age. In the oldest monkeys, positivity increased to 94%. Even using a more stringent IgG and IgA double positive criterion to classify monkeys as *H. pylori*-colonized, the trends remain the same and the same age-dependency was observed for the CagA IgG ELISA. Parallel age trends are commonly observed in studies conducted in human populations [27,45]. However, in contrast to humans, younger Rhesus macaques have the same likelihood to have a CagA-positive strain as do the macaques in the oldest group. Comparing double- (44%) and triple- (29%) positive rates, about 67% of the *H. pylori*-positive monkeys were colonized with a CagA + strain. This is comparable to the situation among humans in developing countries [27,45]. In summary, the socially living Rhesus macaques in captivity resemble the pre-modern situation for *H. pylori* colonization. One possible route of acquiring *H. pylori* in younger monkeys is by oral-oral contact [46] with older monkeys. Free-living monkeys and monkeys in captivity have close contact with each other, making it impossible to trace infection routes back to parents, siblings, aunts or playmates, without the ability to genotype the strains. How the Rhesus macaques originally acquired *H. pylori* also remains unclear. Genotyping would help to elucidate whether the strains are of human origin, and whether such strains were introduced into the Rhesus macaque population before or after they were captured in 1938 and brought to Cayo Santiago Island (i.e. carrying an Asian strain). Strain isolation from gastric biopsies and multi-locus sequence typing (MLST) analysis could help answer these questions.

To determine the cut-off values for the *C. jejuni* ELISA, we used blood samples from humans of known *C. jejuni* status [28]. The tested 94 monkeys did not show any signs of diarrhea at the time of sampling and had no evidence of acute infection. In contrast to *H. pylori*, there was no correlation between age and *C. jejuni* sero-positivity. In general, the prevalence of *C. jejuni* IgG was equally high in all the groups, with a significant drop of prevalence of IgG antibodies in juvenile Rhesus macaques. However, a high prevalence of *C. jejuni* IgA was observed in that group. In areas in which *C. jejuni* infection is hyper-endemic and infection is recurrent, young children develop high levels of specific serum IgG antibodies. With continued exposure, IgG levels wane and IgA levels rise [29,30], and the duration of colonization diminishes reflecting development of gut immunity [36]. The drop in IgG between infant and juvenile macaques that we observed is consistent with this phenomenon. As in humans, *C. jejuni* causes transient infection in Rhesus macaques and specific antibodies are detected in convalescence [47].

## Conclusions

This study describes the distribution of *H. pylori* and *C. jejuni*-specific antibodies in a social group of Rhesus macaques living in captivity. The study indicates that Rhesus macaques have widespread exposure to two important human mucosal bacteria, suggesting that they may provide a good model to study short- and long- term effects of *C. jejuni* and *H. pylori* colonization, respectively, in a population. The ELISAs that were established provide methods to determine the *H. pylori* colonization status and prior exposure to *C. jejuni* in Rhesus macaques.

## Methods

### Animals studies

Two cohorts of Rhesus macaques were studied. Cohort 1 consisted of 30 domestic male Rhesus macaques (*Macaca mulatta*) [2 to 7 years old and weighing 3–5 kg] from the animal facility at the Uniformed Service University of the Health Sciences in Bethesda, Maryland. Samples were collected between January 1988 to May 1995. Upon arrival to the facility, these monkeys had been quarantined for 90 days in individual stainless steel cages in conventional holding rooms of the animal facility [approved by The American Association for Accreditation of Laboratory Animal Care (AALAC)] and were subsequently kept in similar individual cages. Animals were provided with tap water ad libitum, commercial primate chow, and fruit. All subsequent studies were performed after an overnight fast, between 8 am and noon.

The studied social group of monkeys (Cohort 2) consisted of 94 Rhesus macaques housed in the Caribbean Primate Research Center in Sabana Seca, Puerto Rico. These animals are descendants of monkeys brought to the Research Center in 1984 from the free-ranging colony on Cayo Santiago Island. They are co-housed and are in constant contact with one another. Serum samples were collected between December 2008 and April 2010. The studies were performed in a cross-sectional design for determination of *H. pylori* and *C. jejuni* status, according to age. The Rhesus macaques (Cohort 2, N = 94, 60 females, 34 males) were assigned to groups according to age: group 1 (Infant) includes monkeys <1 year [N = 32 (17 females/15 males)]; group 2 (Juvenile) monkeys were 1.0-2.9 years [N = 24(18/6)]; group 3 (Young Adult) monkeys were 3.0-9.9 years [N = 21(12/9)] and group 4 (Adult) consisted of all monkeys ≥10 years of age [N = 17(13/4)]. Of the 32 monkeys in group 1, five (3 female, 2 male) <0.5 years were excluded from serological analysis, due to potential maternal antibodies.

### Endoscopic procedures and biopsies

The 30 Rhesus macaques of Cohort 1 underwent gastroduodenal endoscopic examination under general anesthesia, essentially as described [13]. From each animal, six pinch biopsies of the gastric corpus and six from gastric antral mucosa were obtained. Two biopsies from each region were fixed in neutral 10% buffered formalin and embedded in paraffin. Biopsy sections were stained with haematoxylin and eosin or Genta’s method and viewed under 100x to 1,000x magnification. The presence of inflamamatory cells in the biopsies was scored on coded slides, as described [47]; as were the presence of stained *H. pylori* organisms, as described [13]. Two other biopsies from antrum and corpus were immediately placed in 0.1 ml sterile 0.9% NaCl on ice and processed for *H. pylori* isolation, as described [47]. *H. pylori* isolates were identified as pinhead-colonies, urease-, oxidase- and catalase-positive, and were Gram-negative curved or “gull-wing” rods.

### Measurement of *H. pylori*-specific antibodies in Cohort 1

From each Rhesus macaque at the time of each endoscopy, five milliliters of blood was collected in tubes containing 10.5 mg of EDTA and centrifuged; the supernatant plasma was frozen at −70°C. The monkey samples were diluted 1:800 for IgG and 1:100 for IgA. Anti-*H. pylori* immunoglobulin G (IgG) and A (IgA) levels in the plasma were determined, using a modification of a method with high sensitivity and specificity for human *H. pylori* positivity, as described [13,41]. In brief, the *H. pylori* antigens, composed of a mixture of protein and lipopolysaccharides from five *H. pylori* strains (ATCC 53722, 53721, 53725, 53726, and 53727), were obtained by water extraction and sonication, as described [41]. The mixture was diluted in 0.05 M carbonate buffer (pH 9.6) to coat each well of a flat-bottom ELISA-plate with 1.0 mg antigen. Plates were coated overnight at 4°C and then blocked for 3 h with 1x PBS containing 0.05% Tween-20, 0.1 mg/ml thimerosal, and 0.1% gelatin. Serum samples were added and plates were incubated for 1 h at 37°C. All washing steps were performed with 1x PBS containing 0.05% Tween-20 and 0.1 mg/ml thimerosal. Goat anti-monkey IgG (gamma chain) or IgA (alpha chain) conjugated to horseradish peroxidase (Rockland Immunochemicals Inc., Gilbertsville, PA) was used as second antibody for detection of responses. A cut-off value was established to distinguish between positive and negative results in the IgG enzyme-linked immunosorbent assay (ELISA) by determining ODR-values (optical density ratio). ODRs were determined for each sample by dividing the OD-value of the sample by the OD-value of two positive controls for *H. pylori* and *C. jejuni* IgA and IgG and one positive control for *H. pylori* CagA, which were included on each ELISA plate as reference specimens. Using 9 Rhesus macaques found to be *H. pylori* negative by tissue examination (negative culture or histology), the mean ODR-value plus 3 intervals of standard deviation (SD) from those animals was used to define the threshold for negativity (0.34). For IgA, the mean value of 9 uninfected monkeys plus 2 intervals of SD was used to define the threshold for negativity (0.44). An ELISA to detect anti-CagA IgG in plasma from the monkeys was performed using a purified recombinant CagA antigen, as described [48]. The assay was modified with the use of a goat anti-monkey IgG (gamma chain) conjugate. The monkey plasma was diluted 1:100. A cut-off value was established to distinguish between positive and negative CagA results based on the 9 monkeys found by tissue exam to be *H. pylori*-negative. The mean value plus 2 intervals of SD for the 9 animals was used to define the threshold for positivity (0.20).

### Measurement of *H. pylori*-specific antibodies in Cohort 2

For cohort 2, ELISAs were performed as described above. Each sample was tested twice and in the case of disparate results, a third measurement was performed. If samples had discordant ODR-values in the two runs (values close to the negative and positive controls, respectively), we assumed that at least one value was an artifact, and the assay repeated. An assay also was repeated when the ODR-values were close to the threshold values*.* Resulting ODRs were averaged and evaluated using the determined cut-off values from the reference values from Cohort 1. Additionally, Cohort 2 values were statistically analyzed to evaluate whether the same cut-off values could be obtained without using the reference group. To obtain the cut-off for positive samples, the mean of ODRs between 0.4-1.0 was calculated and 2 times the SD was subtracted, providing a cut-off for positivity (a summary of all values is provided in Table 3). To determine the negative cut-off, the mean of ODRs between 0.000-0.299 was calculated and two times the SD was added. Values between 0.300-0.399 were only excluded for the statistical analysis of the cut-off values. After establishing the cut-off values for positivity and negativity, ODRs between 0.300-0.399 were assigned according to these values.

### *C. jejuni* ELISAs

Since antigens from sonicated whole *C. jejuni* cells did not yield reliable results for serology (not shown), the McCoy antigen [49] (from PEN1, 2, and 3 strains), as described [49,50], was used for IgG and IgA determination. In brief, *C. jejuni* cells were harvested in sterile water, washed twice with water and 0.1 g of wet cells were suspended in 2.5 ml 0.2 M glycine-hydrochloride buffer (pH 2.2). Suspensions were stirred at 25°C for 15 min and centrifuged at 11,000 x g for 15 min. The supernatant was collected and sodium hydroxide was added to neutralize the suspension. Next, the suspension was dialyzed against water for 24 h at 4°C and the protein concentration was determined. ELISA was performed as described for *H. pylori.* Monkey sera were diluted 1:200 and goat anti-monkey IgG (gamma chain) or IgA (alpha chain) conjugated to horseradish peroxidase (Rockland Immunochemicals Inc., Gilbertsville, PA) (1:2000) was used as the secondary antibody. OD_405_ values were normalized using a known positive human control serum (1:200) to compare ODR. Each sample was tested 2–3 times, as described above.

### Determination of cut-off values for *C. jejuni* IgG ELISAs

To initially determine a range to examine seropositivity, the mean of sera with ODRs between 0.000-0.299 was calculated and 2 intervals of SD were added, obtaining an initial negative cut-off of 0.338. Next, the mean of ODRs between 0.4-1.0 was calculated and 2 SD intervals substracted resulting in a positive cut-off of 0.318. We also used 10 serum samples from *C. jejuni*-positive persons and 16 serum samples from *C. jejuni*-negative persons. The calculated negative cut-off was 0.362 and the positive was 0.418. We calculated the mean value for these three tentative cut-offs, and established a cut-off of 0.340 as an alternative means to define the thresholds for positivity and negativity.

### Determination of cut-off values for *C. jejuni* IgA ELISAs

IgA cut-offs were calculated as described for IgG. Since the negative cut-off was 0.368 and the positive cut-off was 0.326, we used a cut-off of 0.350 to analyze Cohort 2.

## Competing interests

The authors declare that they have no competing interests.

## Authors’ contributions

SK participated in the study design, carried out experiments, analyzed the data and drafted the manuscript. GPP carried out experiments and analyzed the data from cohort 1. JLRC and RTA performed immunoassays and helped to analyze the data. HL participated in the statistical analysis of the data. AD created and designed the study and analyzed the biopsies, JAGM provided plasma samples. MGDB and MJB participated in the design of the study and helped to analyze the data and to draft the manuscript. All authors read and approved the final manuscript.
